# Diagnostic challenges and pockets of susceptibility identified during a measles outbreak, Luxembourg, 2019

**DOI:** 10.2807/1560-7917.ES.2021.26.22.2000012

**Published:** 2021-06-03

**Authors:** Michel Kohnen, Patrick Hoffmann, Caroline Frisch, Emilie Charpentier, Aurélie Sausy, Judith M Hübschen

**Affiliations:** 1 Clinical laboratory, Centre Hospitalier de Luxembourg, Luxembourg, Grand Duchy of Luxembourg; 2 Université Libre de Bruxelles, Brussels, Belgium; 3 Health Directorate Luxembourg, Division de l’inspection sanitaire, Luxembourg, Grand Duchy of Luxembourg; 4 Department of Infection and Immunity, Luxembourg Institute of Health, Esch-sur-Alzette, Grand Duchy of Luxembourg

**Keywords:** measles, outbreak, Luxembourg, diagnosis, elimination

## Abstract

Luxembourg was among the first countries in the World Health Organization (WHO) European Region documenting interruption of endemic measles transmission, but an increased incidence was registered in spring 2019. The outbreak started with an unvaccinated student who had been to a winter sports resort in a neighbouring country, where a measles outbreak was ongoing. Subsequently, 12 secondary and two tertiary cases were confirmed among students from the same school, relatives and healthcare workers, as well as six probably unrelated cases. Only 11 cases initially fulfilled the WHO definition for suspected measles cases. Fourteen of 20 cases with information on country of birth and the majority of unvaccinated cases (10/12) were born outside of Luxembourg. Measles IgM antibody results were available for 16 of the confirmed cases, and five of the eight IgM negative cases had been vaccinated at least once. All 21 cases were PCR positive, but for three previously vaccinated cases with multiple specimen types, at least one of these samples was negative. The outbreak highlighted diagnostic challenges from clinical and laboratory perspectives in a measles elimination setting and showed that people born abroad and commuters may represent important pockets of susceptible people in Luxembourg.

## Background

Measles is a highly contagious infectious disease caused by measles virus. The virus belongs to the *Paramyxoviridae* family and is transmitted mainly through respiratory droplets and secretions. Measles virus shedding from the nasopharynx begins before the typical morbilliform rash appears and the virus can survive in the air or on objects and surfaces for up to 2 hours [1]. A cheap and effective vaccine has been available since the 1960s, but coverage rates of ≥ 95% are required to achieve and maintain measles elimination [2].

In Luxembourg, two doses of measles-mumps-rubella and varicella vaccine are recommended at 12 and 15 to 23 months of age, and coverage rates have been estimated at 99% for the first dose since 2014. For the second dose, coverage was estimated at 86% between 2014 and 2017, and at 90% in 2018 [3]. Luxembourg was among the first countries in the World Health Organization (WHO) European Region documenting interruption of endemic measles transmission [4], and in 2015, measles elimination was declared based on data from 2012 to 2014 [5]. Only a few sporadic cases were reported in the years that followed, until an increased disease incidence was noticed in spring 2019, in the context of a concurrent measles resurgence in Europe [6].

## Outbreak detection

In mid-March 2019, several clinical samples from a hospitalised patient were received at the WHO European Regional Reference Laboratory for Measles and Rubella in Luxembourg (RRL-Lux) and all of them tested positive for measles virus RNA. The case was reported to the Division de l’inspection sanitaire (DIS) of the Health Directorate of the Luxembourg Ministry of Health by the physician in charge. The case follow-up revealed that the patient had visited a winter sports resort abroad with many other students who attended the same school. A measles outbreak was ongoing at the resort, with a total of 55 reported cases [7]. Subsequently, several measles cases were linked to this index case, but a few unrelated cases were also identified in Luxembourg.

This article describes the measles cases, the epidemiological and laboratory investigations and the follow-up and control measures implemented to highlight the challenges encountered and lessons learnt.

## Methods

### Case definition, reporting and classification

In Luxembourg, suspected measles cases are defined as patients with the following symptoms: fever, maculopapular rash and one of cough, coryza or conjunctivitis [8], or patients for whom the treating doctor suspects or considers measles virus infection. Measles cases need to be reported by the treating doctor and the diagnostic laboratory confirming the case to the DIS, which is in charge of national infectious disease surveillance. Cases are considered laboratory confirmed if they have a positive IgM and/or PCR result. According to their likely place of infection, measles cases are classified as imported, importation related or with unknown source [9].

### Epidemiological investigation

The epidemiological case follow-up was done by the DIS using a case investigation sheet developed in collaboration with the RRL-Lux. In addition to personal and clinical information, data about occupation, travel at the time of infection and during the incubation time, contact history, vaccination status, etc. were recorded.

### Sample collection and laboratory testing

Throat and/or nasal swabs and, if the delay between rash onset and notification was more than 5 days, oral fluid samples were requested for viral RNA detection; serum was obtained for antibody detection. Serum samples received at the RRL-Lux were tested for measles-specific IgM antibodies using the Enzygnost Anti-Measles-Virus/IgM kit (Siemens, Marburg, Germany), according to the manufacturer’s instructions. Sera of cases with past vaccination were also tested with the Anti-Measles Virus ELISA (IgG) avidity kit (EUROIMMUN, Lübeck, Germany), following the instructions provided in the manual to assess potential vaccine failures. Clinical samples for PCR were submitted to RNA extraction using the QIAamp Viral RNA Mini kit (QIAGEN, Hilden, Germany) and the eluate was tested for measles-specific RNA using two different assays and previously published primers [10-12]. All samples that tested negative for measles IgM antibodies or measles RNA were tested for rubella. Extracted RNA from measles and rubella PCR-negative cases was also checked for an RNAse P signal to verify sample quality. Molecular characterisation of measles virus strains was based on the 450 nt of the nucleoprotein gene routinely used for genotyping [13] and the non-coding region between the matrix and the fusion genes (MF-NCR [14]). Phylogenetic analyses were based on the Kimura 2-parameter model or the number of nt differences and the Neighbour-Joining method using MEGA7 [15]. The sequences were submitted to the Measles Nucleotide Surveillance (MeaNS) database [16] under accession numbers 136859–136861, 136870–136872, 136893, 136895–136897, 136899, 136901–136903, 136906–136908, 138040, 138533 and 139929.

### Ethical statement

The results described herein were obtained as part of routine case and outbreak investigations and thus no ethical approval was needed. Personal information linked to case identifiers is restricted as much as possible to avoid identification.

## Results

### Outbreak description

**Figure 1:**
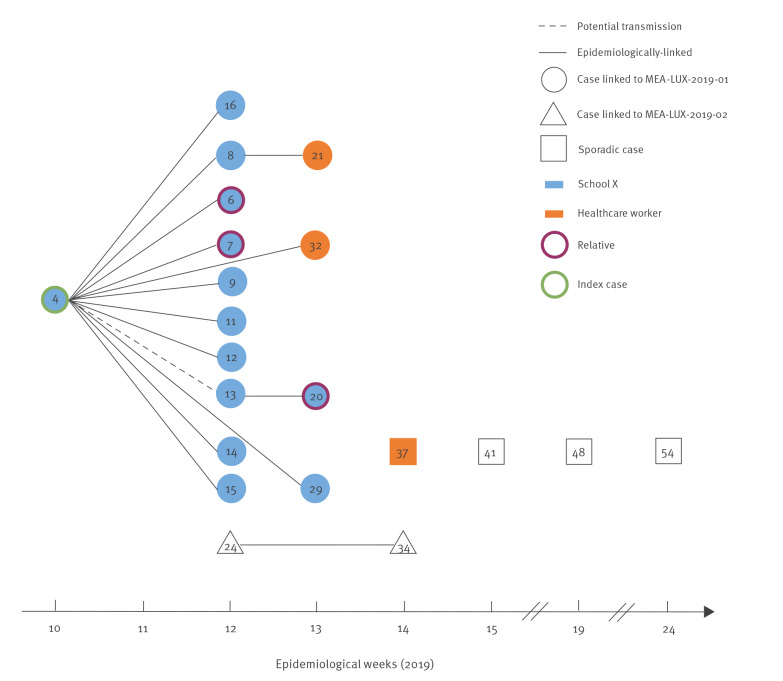
Measles cases by epidemiological week, Luxembourg, 2019 (n = 21)

MEA-LUX-2019-01: the first measles outbreak identified in 2019 in Luxembourg; MEA-LUX-2019-02: the second measles outbreak identified in 2019 in Luxembourg; School X: the school attended by the index case and cases in blue.

The index case in the measles outbreak MEA-LUX-2019–01 was an unvaccinated student (LUX19-H-4) who developed a morbilliform rash during epidemiological week 10, in March 2019 (Figure 1). In addition to the rash, they presented with fever, conjunctivitis/coryza/cough, Koplik spots, fatigue and apathy, which led to hospitalisation. The anamnesis revealed that the case had stayed abroad at a winter sports resort in epidemiological week 8, where a measles outbreak was ongoing. In week 12, 10 secondary cases were detected among family members (LUX19-H-6 and LUX19-H-7) and students from the same school (Figure 1). Two additional secondary cases were registered in week 13, one in a vaccinated healthcare worker (LUX19-H-32, Figure 1) who had been in contact with the index case in hospital. Only two tertiary cases occurred, one in an unvaccinated family member (LUX19-H-20) and the other in a vaccinated healthcare worker (LUX19-H-21, Figure 1). In addition, an unvaccinated person (LUX19-H-24) without any known epidemiological link to the index case was hospitalised in week 12 and led to one secondary case two weeks later (LUX19-H-34, MEA-LUX-2019–02, Figure 1). In week 14, another case was registered in a vaccinated healthcare worker from a neighbouring country, but no epidemiological link to any of the previous cases could be established and exposure occurred most likely outside Luxembourg (LUX19-H-37, Figure 1). Another three sporadic cases were registered in weeks 15, 19 and 24 (Figure 1), and two of these were reported in neighbouring France. Case follow-up was done by the French and Luxembourg authorities, since the concerned individuals were commuters, and laboratory investigation took place at the RRL-Lux.

Case numbers relate to suspected cases notified to Division de l’inspection sanitaire in 2019. Only confirmed measles cases are displayed in the figure. All healthcare workers were fully vaccinated.

### Case characteristics and classifications

Between February and mid-July 2019, the RRL-Lux received samples from 47 of the 48 suspected measles cases notified to the DIS. In three individuals with mild symptoms, the vaccine strain was detected and measles wild-type infection was confirmed in 20. An additional case tested positive in another diagnostic laboratory in Luxembourg, resulting in 21 wild-type infections and an incidence of 3.4 per 100,000 inhabitants. Among the 21 confirmed measles cases, 10 were female and ages ranged from 8 to 48 years (median: 17 years). Three cases were hospitalised with either fatigue and apathy or arthralgia or stomatitis, while five other cases reported symptoms such as adenopathy, headache and throat or gum pain. The most frequently recorded clinical symptoms among the 20 cases with available information were fever (n = 17), rash (n = 14) and cough (n = 13), followed by coryza (n = 8), conjunctivitis (n = 7) and Koplik spots (n = 6). About half of the cases (n = 11) fulfilled the WHO definition for suspected measles cases. Only six of the 20 cases for whom this information was available were born in Luxembourg, while the others originated from various European countries including Italy (n = 5), France (n = 3), Germany (n = 2), Belgium (n = 1), Portugal (n = 1), Romania (n = 1) and Switzerland (n = 1). The majority of the cases were unvaccinated (n = 12) or had only received a single dose of measles-containing vaccine (n = 2); vaccination records were available and checked for six of the vaccinated cases. Ten of the 12 unvaccinated cases were born abroad. Three of the 21 cases had their permanent residence in neighbouring countries outside of Luxembourg. According to the likely place of infection, five of the cases were classified as imported and 14 as importation-related; for the remaining two cases, the status was unclear.

### Laboratory results

All 21 cases were PCR positive, and for 13 cases more than one specimen type was available for testing at the RRL-Lux (Table 1). It was not always the same specimen type that showed the earliest signal in the real-time PCR and for three patients at least one of the specimens collected was negative (Table 1). None of the 24 suspected but discarded cases tested positive for rubella IgM antibodies or rubella RNA, while all of their clinical specimens collected for PCR testing showed an RNase P signal.

**Table 1:** Measles virus diagnostic PCR results according to specimen type, Luxembourg, 2019 (n = 21)

Case identifier	Throat swab	Nasal swab	Oral fluid
Case 1	**+**	**+**	**+**
Case 2^a^	**+**	NA	NA
Case 3^a^	**+**	NA	NA
Case 4^a^	**+**	NA	NA
Case 5	**+**	**+**	NA
Case 6	**+**	**+**	NA
Case 7	**+**	**+**	NA
Case 8	**+**	**+**	**+**
Case 9	**+**	ND	NA
Case 10	NA	**+**	NA
Case 11	**+**	ND	ND
Case 12	**+**	**+**	NA
Case 13	**+**	ND	NA
Case 14	**+**	**+**	**+**
Case 15^b^	NA	NA	NA
Case 16^c^	NA	NA	NA
Case 17	**+**	**+**	NA
Case 18^a^	**+**	NA	NA
Case 19	NA	**+**	**+**
Case 20	**+**	**+**	**+**
Case 21	**+**	NA	NA

+: positive; NA: not available; ND: not detected; RRL-Lux: WHO European Regional Reference Laboratory for Measles and Rubella in Luxembourg.

^a^ Dry throat swabs were sent to the RRL-Lux.

^b^ No sample was sent to the RRL-Lux.

^c^ Sample was sent to the RRL-Lux for genotyping only.

The specimen type with the earliest signal in the real-time PCR is highlighted in green; the PCR negative results are highlighted in orange.

Case numbers not identical with those in Figure 1.

**Figure 2:**
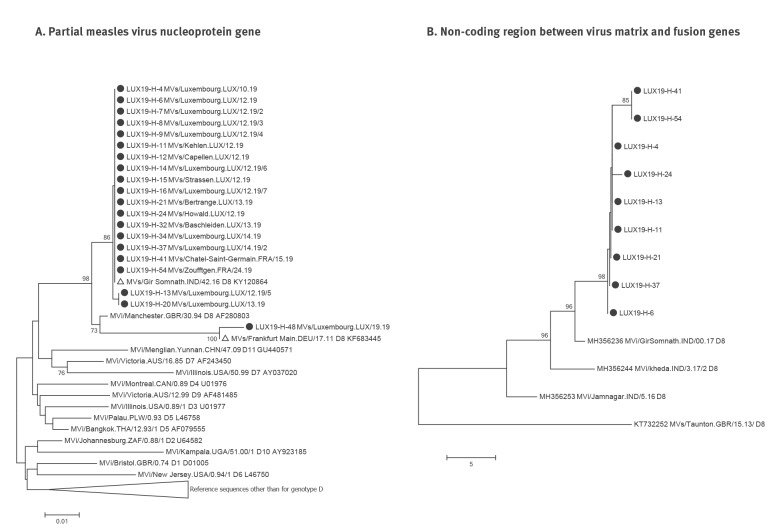
Phylogenetic tree based on (A) 450 nt of the measles virus nucleoprotein gene and the Kimura 2-parameter model and (B) the non-coding region between the measles virus matrix and fusion genes and the number of nt differences

Genotype data are available for all 20 measles cases for which original material was received at the RRL-Lux. The measles viruses belonged to three different sequence variants of genotype D8, with the Gir Somnath variant being by far the most abundant (n = 17, Figure 2A). Virus sequences from Cases 13 and 20 differed by one nt from the Gir Somnath variant. The virus sequence isolated from Case 48 was clearly distinct from the other two variants and was most similar to the Frankfurt Main strain (Figure 2A). Overall, nine full-length MF-NCR sequences were obtained, which differed by up to 3 nt in the 1018 nt region (Figure 2B). The sequences of cases LUX19-H-41 and LUX19-H-54 differed by 2 nt from the variant found in six of the nine samples, and the sequence of one of the two cases with unknown source of infection (LUX19-H-24) differed by one nt from that variant (Figure 2B).

Measles IgM antibody results were available for 16 of the confirmed cases, seven of which were positive and one equivocal. Five of the seven IgM positive patients reported a rash and the sample collection took place between 1 day before and 4 days after rash onset. Except for one patient who had received two doses of measles-containing vaccine, all IgM positive cases were unvaccinated. Five of the IgM negative patients had been vaccinated at least once and, for the three IgM negative patients with rash onset date information, the samples had been collected within 2 days of rash onset. Of the nine patients who did not meet the WHO clinical case definition at diagnosis, two were in addition to PCR also confirmed by IgM detection; in addition, three were unvaccinated, two had received one dose of vaccine and the remaining four had received at least two doses. For two of the nine cases with past vaccination, it is known that they met the measles clinical case definition at diagnosis and one of the seven cases with IgM results tested positive (Table 2). Of the three vaccinated cases for which there were sera available for IgG avidity testing, two showed a high avidity, suggesting previous contact with measles virus, and one had too low a level of IgG antibodies for any conclusive avidity assessment.

**Table 2:** Clinical symptoms at diagnosis and IgM test results for measles outbreak cases previously vaccinated against measles virus, Luxembourg, 2019 (n = 9)

Symptoms	Vaccinated case no. (no. of doses)								
	Case 1 (1)^a^	Case 2 (1)	Case 3 (2)	Case 4 (2)	Case 5 (2)	Case 6 (2)	Case 7 (2)	Case 8 (2)	Case 9 (3)
Fever	NR	R	NR	NR	R	NA	R	R	R
Rash	NR	NR	R	R	R	NA	NR	R	R
Cough	NR	R	NR	NR	NR	NA	R	R	NR
Coryza	NR	NR	NR	R	R	NA	NR	R	NR
Conjunctivitis	NR	NR	NR	R	R	NA	NR	R	NR
**IgM results**	**Neg**	**Neg**	**Neg**	**NA**	**Equ**	**Neg**	**Pos**	**NA**	**Neg**

Equ: equivocal; NA: information not available; no.: number; NR: not reported; Neg: negative; Pos: positive; R: reported.

^a^ Case was investigated because of close contact with a confirmed measles case.

Case numbers not identical with those in Figure 1.

## Outbreak control measures

The DIS initiated an investigation of all suspected measles cases within 24 hours after notification. Both the doctor in charge and the patient were contacted during the case follow-up.

The MEA-LUX-2019–01 outbreak comprised 15 confirmed measles cases. A total of 698 people had confirmed or possible contact with at least one of the outbreak cases during their infectious period (5 days before symptom onset or test result if asymptomatic), and were traced to the school the index case attended, three different sports clubs and family member and friend groups. These people were contacted by letter, email or phone. In addition, an information sheet, which did not mention any names, was distributed via the school health service and the sports club management, with information regarding the symptoms of the disease and a request to immediately contact a doctor or the DIS in case of any symptoms compatible with measles. Confirmed or possible contacts were advised to monitor themselves until 18 days after reception of the information about the outbreak. Vaccination cards (if available) were checked by the school health service or the DIS. Nearly 70% of the contacts (n = 491) had complete immunisation records, with two doses of measles-containing vaccine, while 58 persons had documentation of only one dose and 149 had not received any measles vaccine or were not aware of their vaccination status. For all people with missing information or incomplete vaccination status, measles vaccination was recommended. However, it remains unclear how many people took advantage of the free-of-charge vaccine offered by the Health Directorate in the outbreak context.

Reaching out to the 698 confirmed or possible contacts allowed for detection of not only mild cases with incomplete clinical presentation, but also some new cases on the first day of symptom onset, which facilitated a timely case investigation. Most of the confirmed cases were put under quarantine, although this measure was not followed in all cases.

The population was informed about the measles outbreak via press releases sent by the Health Directorate and was urged to have their vaccination cards checked and get vaccinated according to the national recommendations. People were also encouraged to contact the DIS in case of any questions about measles or measles vaccination and, according to the DIS, there was indeed an increased demand for information on this topic.

## Discussion

The measles outbreak in Luxembourg in spring 2019 originated from an importation from a holiday resort in a neighbouring country with a locally ongoing measles outbreak. It was facilitated by the close proximity of the index case to several secondary cases during the trip and at school, as well as the insufficient vaccination coverage among this group. Not only were 14 of the 20 cases with birth location information born abroad, but also 10 of 12 of the unvaccinated cases were from European countries other than Luxembourg. The measles-containing-vaccine first-dose coverage in Luxembourg has been estimated at ≥ 95% since 2003, while this coverage was 84–93% in Italy and 70–91% in neighbouring France during the same period [17], suggesting important immunisation gaps.

There were three commuters among the cases identified in Luxembourg, raising the question of which country is responsible for case management, follow-up and reporting, and how information exchange is organised so that it is the most efficient. In fact, two of the cases consulted a doctor in their country of residence, and thus were reported in that country, while the Luxembourg authorities handled case follow-up and the laboratory investigation was also done in Luxembourg. With an estimated 200,000 people travelling to Luxembourg every day for work [18], there is a considerable risk of introducing infectious diseases including measles, especially since measles outbreaks were ongoing in all of the countries surrounding Luxembourg in 2019 [6]. In spite of this, the 2019 outbreak was the first to comprise more than two cases since 1996, and probably involved even two tertiary cases. While this might seem to suggest that the public health response was somewhat slower or less efficient than in previous years, this is unlikely, as for each case the investigation was initiated within 1 day of notification and the whole outbreak lasted less than 3 weeks (time from notification of the first case to the last case). During this time 698 confirmed or possible contacts were contacted and counselled. Given the limited number of cases, a massive outbreak response was initiated, similar to what has been described before [19,20]. The outbreak did, however, also identify shortcomings concerning the quarantining of cases and the lack of data on vaccine uptake after the DIS encouraged vaccination and offered it free of charge. Like in other reports [21,22], some healthcare workers were affected during the Luxembourg outbreak, although all of them were fully vaccinated against measles. No onward transmission was noticed, which considerably facilitated outbreak control.

Ensuring that the population was well informed and aware of the outbreak, led to very early detection of some cases, which again favoured outbreak control activities, but also supported identification of very mild cases. In fact, only about half of the measles patients fulfilled the WHO suspected case definition and in some cases even rash or fever were absent. This may, in part, be because of the very early identification of some cases, but probably also because of their vaccination status [23,24], since nine had received at least one dose of measles-containing vaccine. These findings suggest that in highly vaccinated populations—and at least in outbreak situations—a less strict clinical case definition should be considered to avoid missing cases [25,26]. Our findings also confirmed that in an elimination setting, more comprehensive laboratory investigations are necessary [27], as no IgM antibodies were detected in half of the measles cases confirmed by PCR (for whom the information was available) and, for three cases, only one of the clinical specimens collected was PCR positive. The IgM negativity might be related to an early sample collection time i.e. within 2 days of rash onset for the three cases with rash onset date information, and/or the vaccination status of the cases [28], since five of them had been vaccinated at least once. The negative PCR results might be due to suboptimal sample collection, although all related specimens were positive in the RNAse P PCR, and/or the lower viral load found after past vaccination [24]. Indeed, all three patients for whom at least one specimen was PCR-negative, had been vaccinated at least twice. Unfortunately, IgG avidity testing was possible for only three cases with past vaccination and the detection of affinity matured IgG antibodies in two of them suggested waning of protective antibody levels rather than primary contact with measles virus.

The routine genotyping data obtained from the Luxembourg cases were helpful to support the assumption of an independent importation of case LUX19-H-48, but not of cases LUX19-H-37, LUX19-H-41 and LUX19-H-54, which were all infected with the Gir Somnath variant of measles virus genotype D8 responsible for most outbreak-associated cases. However, the MF-NCR region, which had recently been suggested useful to further clarify transmission chains [29,30], showed two nt substitutions for LUX19-H-41 and LUX19-H-54 as compared with the outbreak strains and thus supported the assumption that these cases were not related to the Luxembourg outbreak. Cases LUX19-H-13 and LUX19-H-20 differed by one nt from the Gir Somnath variant in the 450 nt region routinely obtained for genotyping. However, since the index case and case LUX19-H-13 attended the same school and used the same public transport to get there, a link to the outbreak was established and later supported by finding the same MF-NCR sequence in LUX19-H-13 as in other outbreak cases. On the other hand, the sequence of one of the two patients with unknown source of infection (LUX19-H-24) differed by one nt from the outbreak strains in the MF-NCR region, suggesting that there was indeed no link to the Luxembourg outbreak.

In conclusion, the measles outbreak in spring 2019 highlighted diagnostic challenges from a clinical and—perhaps even more importantly—from a laboratory point of view, in a measles elimination setting. Our study suggests that in a highly vaccinated population it is essential to suspect measles even if the clinical case definition is not fully met, to collect different specimen types for a comprehensive laboratory analysis and to make efforts to identify vaccine failures, especially in healthcare workers. The outbreak also showed that despite high national vaccination coverage, people born abroad and commuters may present non-negligible pockets of susceptibility, especially in an employment-offering country with a high level of immigration such as Luxembourg. To identify existing vaccination gaps in the whole population, screening programmes for individuals above the age of routine measles immunisation should be considered and could, for example, be conducted by school health or occupational health services.
